# Development of a novel mammalian display system for selection of antibodies against membrane proteins

**DOI:** 10.1074/jbc.RA120.015053

**Published:** 2021-01-13

**Authors:** Nathan Robertson, Nancy Lopez-Anton, Shalom A. Gurjar, Hena Khalique, Zainab Khalaf, Siobhan Clerkin, Vaughan R. Leydon, Richard Parker-Manuel, Alexander Raeside, Tom Payne, Tim D. Jones, Len Seymour, Ryan Cawood

**Affiliations:** 1 OXGENE, Medawar Centre, Oxford, United Kingdom; 2 Anticancer Viruses and Cancer Vaccines Group, Department of Oncology, University of Oxford, Oxford, United Kingdom

**Keywords:** mammalian display, epithelial cell adhesion molecule (EpCAM), chimeric antigen receptor T cells (CAR-T), therapeutic antibody discovery, antibody, antibody engineering, membrane protein, immunotherapy

## Abstract

Reliable, specific polyclonal and monoclonal antibodies are important tools in research and medicine. However, the discovery of antibodies against their targets in their native forms is difficult. Here, we present a novel method for discovery of antibodies against membrane proteins in their native configuration in mammalian cells. The method involves the co-expression of an antibody library in a population of mammalian cells that express the target polypeptide within a natural membrane environment on the cell surface. Cells that secrete a single-chain fragment variable (scFv) that binds to the target membrane protein thereby become self-labeled, enabling enrichment and isolation by magnetic sorting and FRET-based flow sorting. Library sizes of up to 10^9^ variants can be screened, thus allowing campaigns of naïve scFv libraries to be selected against membrane protein antigens in a Chinese hamster ovary cell system. We validate this method by screening a synthetic naïve human scFv library against Chinese hamster ovary cells expressing the oncogenic target epithelial cell adhesion molecule and identify a panel of three novel binders to this membrane protein, one with a dissociation constant (*K_D_*) as low as 0.8 nm. We further demonstrate that the identified antibodies have utility for killing epithelial cell adhesion molecule–positive cells when used as a targeting domain on chimeric antigen receptor T cells. Thus, we provide a new tool for identifying novel antibodies that act against membrane proteins, which could catalyze the discovery of new candidates for antibody-based therapies.

Mammalian display was originally conceived for affinity maturation of single-chain variable fragments (scFv) expressed on the surface of human cells (1) and has been further developed for screening full-length antibody cell surface–expressed libraries (2, 3). The use of mammalian cell display (4) has some important advantages over other display systems (*e.g.* phage/yeast), in particular in relation to the manufacturability of the identified antibodies; in mammalian display, antibodies are produced using the endogenous eukaryotic secretion machinery enabling correct folding and biophysical properties and are therefore more likely to be compatible with mammalian cell production systems. However, a disadvantage of using mammalian display is that only a relatively small library size (usually up to 10^7^) can be interrogated. The library sizes that are available for mammalian systems are typically limited by low transfection efficiency, although recent advances have improved this, for example by using CRISPR–Cas9 integration methods (5, 6). Alternatively, library size can be effectively expanded by first utilizing phage display (7, 8) to screen much larger naïve libraries before converting to mammalian cell display after one or two rounds of selection or by using libraries derived from immunized animals, in which initial antibody selection and maturation has occurred *in vivo* (6). With current mammalian display methods, the cells displaying the antibodies are incubated with the target antigen, which must be available in a soluble format and used either free in solution or bound to paramagnetic beads (1, 9, 10). The latter system can be advantageous in enhancing antigen avidity, thus allowing for the selection of cells that express low affinity antibodies (11). Because purified antigen must be applied to cells in solution or coupled to particles, the target is typically restricted to proteins or protein domains that are soluble and relatively stable; thus, identifying antibodies to membrane proteins remains challenging. Although whole-cell panning methods can be used with phage display to enrich for phage that bind to complex membrane proteins (12, 13), the phage require reformatting, and this process can frequently ablate binding activity, thereby reducing the number of positive binders for subsequent analysis. This process also nonspecifically enriches for phage against unrelated, nontarget, cell surface proteins.

The technology to screen large naïve libraries within a mammalian setting would clearly confer a significant advantage by improving the compatibility and developability of identified antibodies with mammalian cell manufacturing systems, without the requirement to use two distinct discovery methods. Furthermore, because many important therapeutic targets are membrane proteins, the ability to screen against a membrane antigen in its native configuration within a cellular membrane environment would ensure that only physiologically relevant epitopes are presented, thus giving a greater likelihood of identifying functional antibodies. To provide a mammalian display technique with both these major advantages, we here describe a method by which we package large antibody libraries with diversities of ∼10^9^ into lentiviral particles and use these to transduce CHO cells that have been engineered to express the target membrane protein. This allows much larger library sizes to be sampled than with existing methods (by at least 100-fold) and is only limited by the number of cells that can be cultured in the laboratory. Our strategy results in individual CHO cells expressing the target antigen on their cell surface while co-expressing and secreting a variant from the high diversity scFv library. Thereby, CHO cells that express an scFv variant capable of binding the target undergo self-labeling, thus allowing them to be isolated, reselected, and eventually sequenced. Reported strategies have identified co-expression of scFvs and antigen in a single cell culture as a method to screen for antibodies, for example in a bacterial display system (14) and for affinity maturation in mammalian cells (15). However, we describe for the first time the screening of a large naïve scFv library fully in a mammalian system and identify binders to a membrane protein antigen presented on the cell surface.

We present here data showing the isolation of CHO cells that secrete influenza hemagglutinin epitope (HA)–tagged scFvs that specifically bind to the membrane protein EpCAM. EpCAM represents a type I transmembrane glycoprotein and has been previously identified as a tumor-associated antigen, most notably because of its overexpression in rapidly expanding epithelial tumors (16). In addition, this oncogene has been shown to contribute to several other biological processes including cell migration, cell adhesion, and proliferation (17, 18).

In brief, self-labeling cells were enriched by magnetic activated cell sorting (MACS), a common method used to isolate and purify specific cell populations (19). This was followed by FACS to isolate a cell population that exhibited FRET in the presence of fluorophore-labeled anti-EpCAM and anti-HA antibodies. FRET has previously been used to monitor protein–protein interactions within the context of flow cytometry and thereby facilitates the assessment of interactions in large cell numbers (20). Because of the proximity requirements of the donor and acceptor fluorophores, a FACS-based FRET assay enabled CHO cells expressing specific antigen binding scFvs to be distinguished from those that bound endogenous cell surface proteins. In this way, single cell clones were isolated, screened, and sequenced leading to the identification of novel scFvs that specifically bound to the EpCAM protein on the cell surface. We further demonstrated the utility of the identified scFvs for the targeting of CAR-T cells to EpCAM-positive cells.

## Results

### CHO cell engineering enables self-labeling by co-expressing EpCAM and a known anti-EpCAM scFv

**Figure 1:**
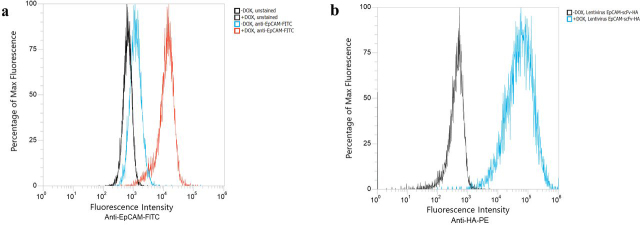
**Flow cytometry analysis of CHO–EpCAM cells.***a*, cells stained with anti-EpCAM FITC in the absence or presence of doxycycline. *b*, cells transduced with lentiviruses expressing a control anti-EpCAM scFv with an HA tag stained with anti-HA-PE antibody in the absence or presence of doxycycline, *i.e.* demonstrating self-labeling.

CHO cells expressing human EpCAM under a doxycycline inducible promoter were generated by transduction of a parental suspension CHO-X cell line with a lentivirus encoding the EpCAM gene and a puromycin resistance marker. The pool of CHO cells then underwent puromycin selection, and cells that showed the highest levels of doxycycline-induced EpCAM expression, determined by surface EpCAM staining, were sorted into single cells by FACS and subsequently expanded (Fig. S1). These clonal cell lines were then screened for EpCAM expression and a high-expressing CHO–EpCAM clone was identified for use in further experiments (data not shown). Doxycycline-induced and noninduced CHO–EpCAM cells were analyzed for EpCAM expression by flow cytometry using an anti-EpCAM FITC-conjugated antibody (Fig. 1*a*). The results showed 92% of cells positive for EpCAM upon induction relative to control cells and uninduced cells showed 39% of cells positive for EpCAM as compared with control cells, suggesting that some baseline expression of EpCAM was present in the uninduced state. CHO–EpCAM cells were then transduced with a second lentiviral construct containing a known anti-EpCAM scFv with a C-terminal HA tag. After culturing for 3 days either in the presence or absence of doxycycline, the cells were stained with an anti-HA–phycoerythrin (PE) antibody, which showed that, although the CHO–EpCAM cells exhibited leaky EpCAM expression in the absence of doxycycline (Fig. 1*a*), a >100-fold increase in self-labeling signal was observed in the presence of doxycycline (Fig. 1*b*).

### MACS enrichment and FRET-based FACS: selection of CHO–EpCAM cells expressing a scFv library that exhibit self-labeling

**Figure 2:**
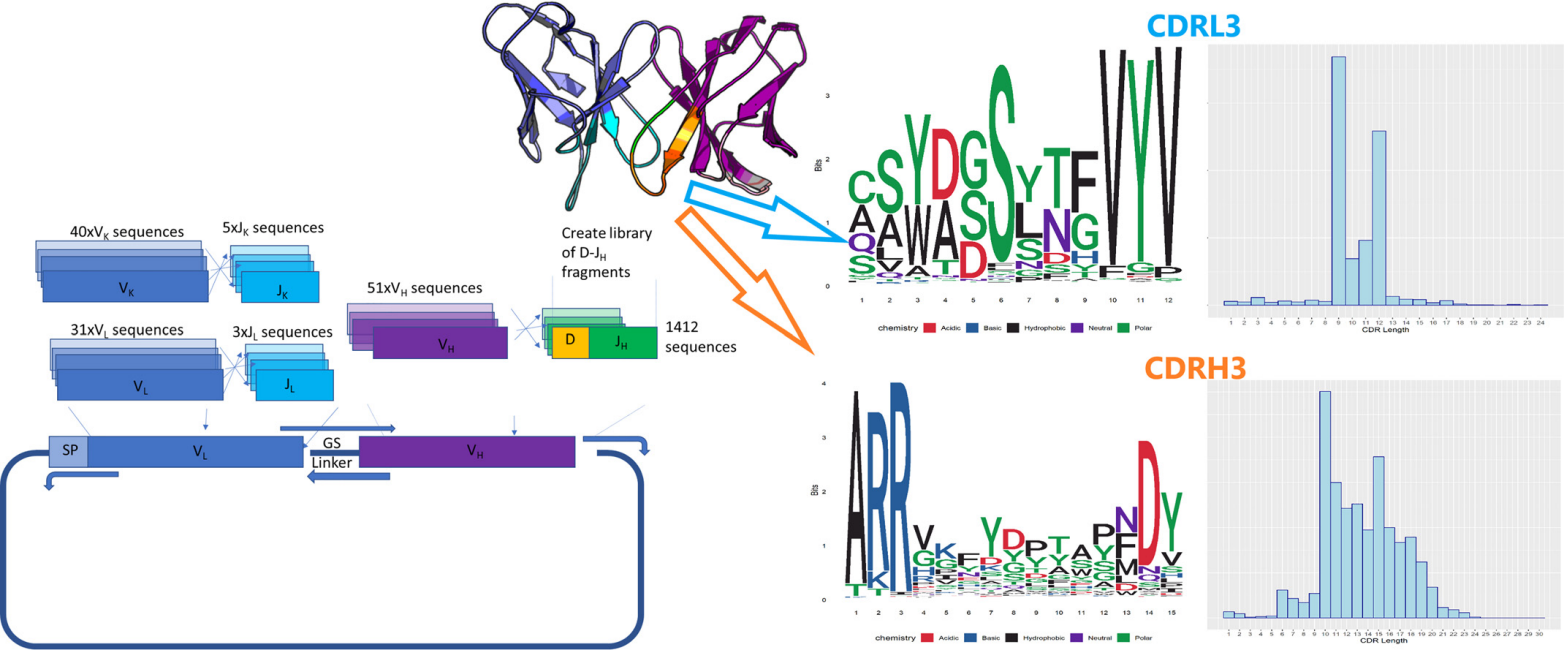
**Schematic overview of the construct of the scFv CHESS library, showing the numbers of V(D)J fragments recombined and shuffled into a lentiviral transfer plasmid.** Natural germ-like variability was observed by NGS analysis of the CDR regions, the most variable being the CDR3 of the light and heavy chain. LOGO plots of the most common CDR3 length from these regions are shown alongside a histogram distribution of CDR3 lengths (*right*).

A large culture (1.5 liters) of EpCAM expressing CHO cells (1.35 × 10^9^ cells) was transduced with a lentivirus preparation packaging an untrained scFv library at a multiplicity of infection (MOI) of 1. The scFv library used was generated by shuffling of synthesized antibody fragments comprising the human germline repertoire of V, D, and J domains of the heavy chain and of V and J domains of the light chain. This synthetic library, named CHESS (CDRs from Human Efficiently Shuffled to form scFvs), is therefore a synthetic re-creation of the human naïve repertoire and comprises a natural-like repertoire of framework regions and diversity within the CDRs in terms of both sequence and length (Fig. 2). The theoretical maximum diversity of the library was 8.6 × 10^8^ and was experimentally determined to contain ∼6.0 × 10^8^ variants by counting colony-forming units by limited dilution of transformants containing the lentiviral packaging plasmid used for lentiviral preparation.

**Figure 3:**
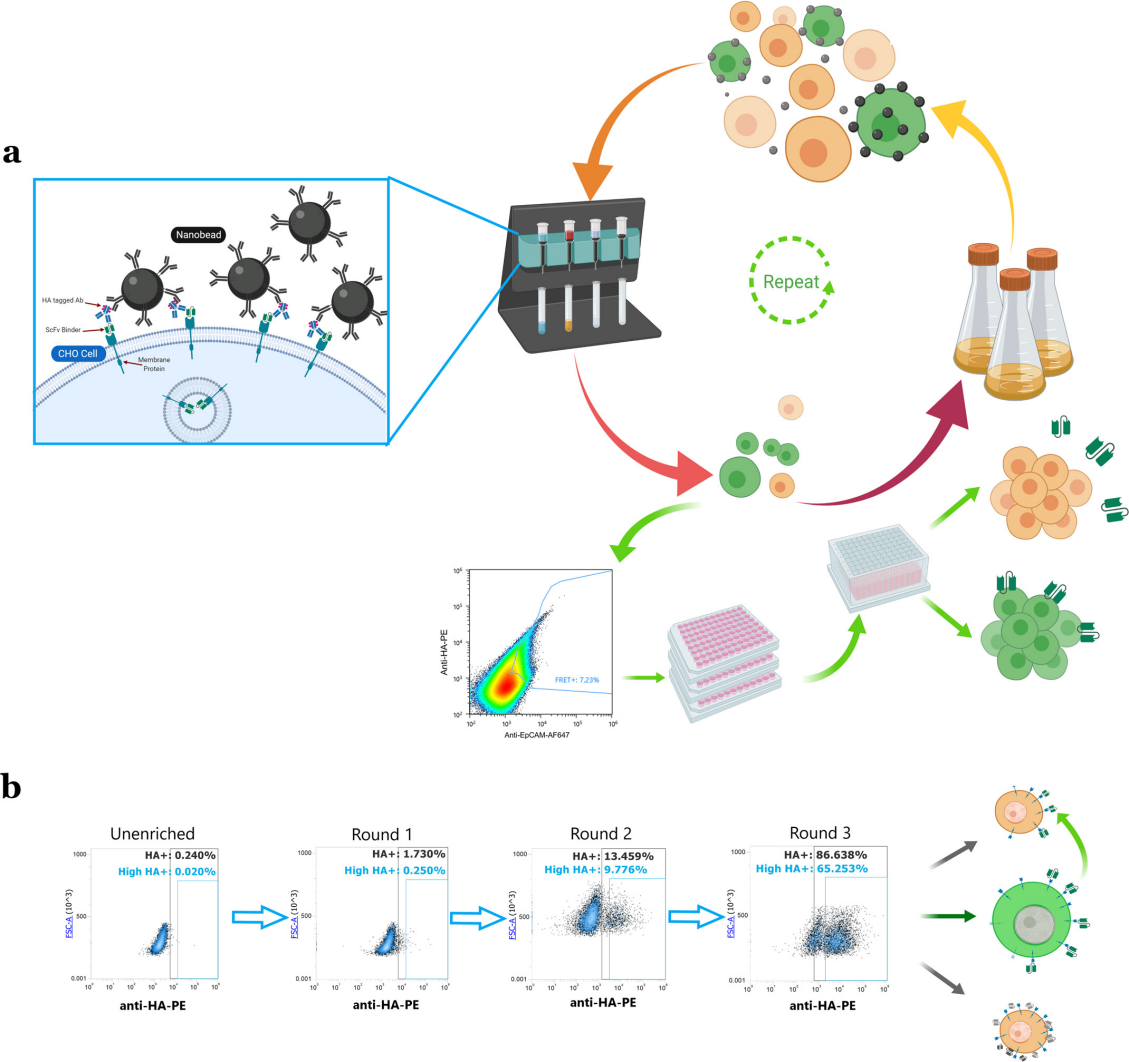
*a*, schematic of nanobead labeling, and overview of selection procedure. Images created with BioRender. *b*, flow cytometry scatter plots of cell outputs from three rounds of MACS selections showing anti-HA-PE staining. The enriched CHO cell culture contains three populations as shown in the schematic (*right panel*): cross-labeled cells, off-target self-labeled cells and target antigen-specific self-labeled cells.

To select for the cells expressing potential EpCAM binders, the scFv library transduced cells were cultured for 4 days with doxycycline to induce EpCAM expression and subsequently subjected to three rounds of enrichment by MACS using an automated system (AutoMACS, Miltenyi Biotec). In each round, the cells were incubated with PE-labeled anti-HA tag antibodies prior to decoration with anti-PE–coated MACS MicroBeads and capture on magnetic columns. The positive cell fraction was eluted from the magnetic columns and allowed to recover for 3–4 days before repeating EpCAM induction and MACS selection (Fig. 3*a*). In the first round, the self-labeled CHO–EpCAM cells were captured from a total cell population of 4 × 10^9^ cells with an output of 4.2 × 10^7^ cells. After allowing the cells to recover, 7 × 10^8^ cells were labeled and selected as before with an output of 2.0 × 10^7^ cells. For the third round of MACS selection, 1 × 10^8^ cells were labeled and selected with an output of 7.6 × 10^6^ cells. After three rounds of selection, the HA-staining positive cell fraction was 86% (Fig. 3*b*).

At this stage, the enriched cell fraction was likely to contain three populations: the intended CHO–EpCAM population that had undergone self-labeling by EpCAM-specific scFv (self-labeling), cells labeled by EpCAM-specific scFvs secreted by neighboring cells (cross-labeling), and cells labeled by scFvs specific for off-target endogenous CHO cell surface proteins (off-target labeling). Therefore, FACS-based strategies were designed to specifically enrich for cells self-labeled with EpCAM-specific scFvs as follows.

**Figure 4:**
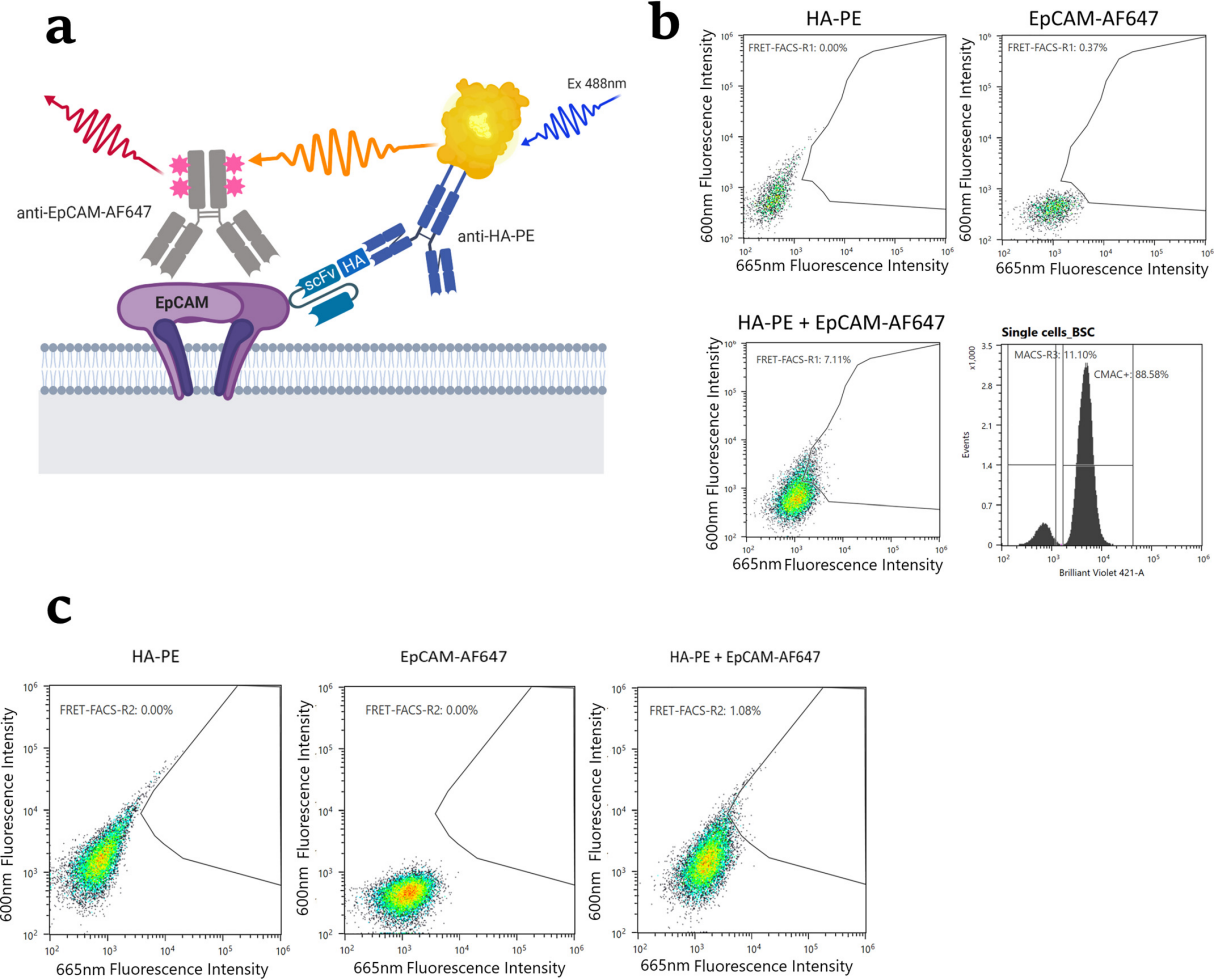
**FRET analysis of MACS-enriched cell pools after three rounds of selection.***a*, schematic of the FRET generation between the donor and acceptor fluorophores upon antigen binding by an scFv library member. *b*, scatter plots of the MACS-enriched CHO cells labeled with anti-HA-PE only (*top left panel*), anti-EpCAM-AF647 only (*top right panel*), and double labeling to generate FRET signal (*bottom left panel*). The histogram plot (*bottom right panel*) shows MACS-enriched cells separated from CMAC stained decoy cells (cells mixed at a ratio of 1:9). *c*, scatter plots showing the sorting gate used in the second round of FACS. The *left* and *middle panels* show the single stained controls, whereas the *right panel* shows the double stained (sorted) cell sample.

To minimize cross-labeling, the cells enriched by three rounds of MACS were co-cultured with an excess (1:9) of “decoy” CHO–EpCAM cells not expressing scFv, *i.e.* the same cell line used for lentiviral library transduction. Thus, when the cell mix was cultured in the presence of doxycycline to induce target expression, the decoy cells would capture the specific and nonspecific scFvs released by the enriched cells. Decoy cells were prelabeled with the live-cell dye CellTracker^TM^ Blue CMAC so that they could be sorted away from the MACS-enriched population. To minimize off-target labeling, a FRET assay was implemented to isolate the cell population that exhibited FRET in the presence of a donor fluorophore, anti-HA–PE, and an acceptor fluorophore, anti-EpCAM–Alexa Fluor 647 (Fig. 4*a*). Because the donor and acceptor fluorophores have to be in close proximity to generate a FRET signal, specific antigen interactions are more likely to generate a signal than off-target interactions; therefore, this method enables distinction of CHO cells exhibiting specific antigen binding scFvs from those that bind endogenous cell surface proteins.

The output from the magnetic enrichments was subjected to two rounds of FACS on an SH800 cell sorter (Sony) by selecting the cells exhibiting a FRET signal in the far-red wavelength (665/30 nm) by proximity of the Alexa Fluor 647 fluorophore to the PE donor fluorophore upon co-binding to the target EpCAM. The cell sample was simultaneously co-cultured with CMAC-labeled CHO–EpCAM decoy cells to reduce the extent of cross-labeling as outlined above (Fig. 4*b*). 5 × 10^6^ MACS-enriched cells were mixed with 4.5 × 10^7^ decoy cells and cultured for 2–3 days in doxycycline-containing culture media. The sort gate was created in a bidimensional plot representing anti-HA–PE (600/60 nm) and anti-EpCAM–AF647 (665/30 nm) fluorescence of double stained cells upon blue laser excitation at 488 nm (Fig. 4*b*). In the first FACS round, 36,000 FRET-positive cells (7.23%) were sorted into a pool and recovered until outgrowth of 6 × 10^6^ cells was achieved. These cells were then sorted a second time, using FRET in the presence of labeled decoy cells, into single cells in 96-well plates (600 cells in total), using a more restrictive gate (1.35%) (Fig. 4*c*), and expanded over a 3-week period.

### Identification of cells secreting positive scFv that are specific to EpCAM

Outgrowth of clones was monitored on a Cell Metric® imaging system (Solentim) coupled to a Microlab STAR robot (Hamilton). The surviving clones were picked on day 18 postsorting and cultured in standard shaking conditions in 96–deep well plates.

CHO–EpCAM clones that survived sorting and outgrowth (*n* = 107) were assessed for their ability to self-label with scFvs in the presence and absence of antigen induction by doxycycline. Induced and noninduced cells were stained with an anti-HA tag PE antibody, followed by flow cytometric analysis (Fig. S2). Of 93 evaluable clones, 26 were selected on the basis of showing a higher signal on doxycycline-induced cells compared with noninduced cells, suggesting EpCAM specificity. These clones were further analyzed using a cross-labeling study, whereby culture supernatants containing secreted scFvs from each clone were tested against target antigen expressing CHO–EpCAM cells and nonexpressing control CHO-X cells (Fig. S3). As a result (21), candidate CHO clones were selected because of their apparent secretion of scFvs specific to EpCAM; 11 clones showed strong binding to CHO–EpCAM cells with no binding to control CHO-X cells and were therefore EpCAM-positive; one clone showed strong binding to both CHO–EpCAM cells and CHO-X cells and was therefore specific for a CHO cell surface protein; the remaining 14 clones showed more variable or weak binding, but an additional 10 clones were selected on the basis of displaying some degree of differential binding between antigen-positive and -negative CHO cells (Fig. S3b).

### Identification of candidate scFv sequences, IgG reformatting, and validation against CHO–EpCAM cells and SPR

From the output of the second round of FACS enrichment, the variable domain genes from the 21 selected EpCAM-positive binders were PCR-amplified and sequenced. As a result, we identified six sequence distinct clones (represented by SP12-E10, SP12-F2, SP14-C8, SP16-E7, SP17-F7, and SP18-C11), four of which were observed in more than one clone, with the sequence of SP12-F2 being represented in eight independently selected clones (SP12-F2, SP14-G8, SP15-B10, SP15-D3, SP15-D9, SP15-E11, SP17-E7, and SP18-F6). Three clones (SP12-E10, SP14-C8, and SP17-F7) shared a common heavy chain sequence but had different light chain sequences.

**Figure 5:**
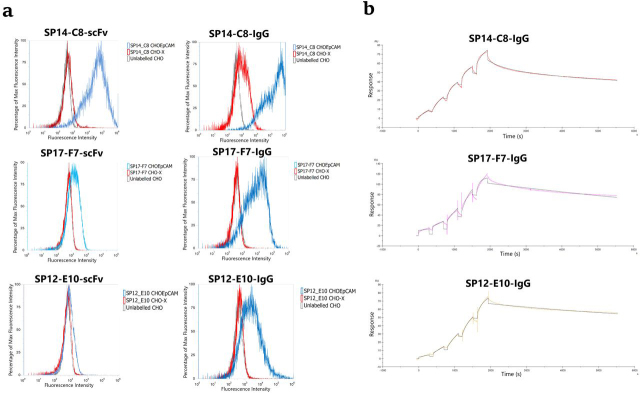
**EpCAM-binding analysis of selected anti-EpCAM sequences that had been recloned and expressed either as scFv or as whole IgG1.***a*, flow cytometry analysis of binding of both scFv (*left panel*) and whole IgG (*right panel*) to CHO–EpCAM cells and CHO-X control cells. *b*, single-cycle kinetics SPR sensorgrams of antibodies formatted as whole IgG1 and captured on protein A sensor chips binding to EpCAM-ECD. The data from the curve fitting is shown in Table 1. SPR traces show representative data from four independent experiments.

All six sequences were recloned as both scFv and full-length IgG1 into expression plasmids and transfected into suspension HEK293 cells modified to express EBNA1 (293OX-EBNA). The supernatants from the transfected cells were once again challenged against CHO–EpCAM cells or CHO-X control cells to confirm their specificity. Only SP12-E10, SP14-C8, and SP17-F7 (Fig. 5*a*) were found to bind specifically to CHO–EpCAM cells when formatted as both scFv and whole IgG1; the SP16-E7 clone exhibited no binding in either format, whereas SP12-F2 or SP18-C11 were found to bind weakly when formatted as scFv but did not bind in the IgG1 format (data not shown). This result with SP12-F2 was particularly surprising because the initial cross-labeling assay results were very clear and positive (Fig. S3), and this sequence had been heavily selected for during panning because it was present eight times in the sequenced clones. The three positive clones displayed different binding characteristics either as scFv or whole IgG1 to the CHO–EpCAM cells with clone SP14-C8 showing the strongest binding in each case. The IgG1 converted clones were then purified via a protein A affinity column and assessed by SDS-PAGE and SEC-HPLC (Figs. S4 and S6). The antibodies were well-expressed and showed only minimal signs of aggregation. These purified IgG variants were then applied to CHO–EpCAM and CHO-X control cells in a titration experiment to determine the EC_50_ on whole cells (Fig. S5). The IgG derived from the clone SP17-F7 revealed only very poor binding at the highest concentrations (5 μm); therefore an accurate EC_50_ could not be determined. However, the IgGs derived from SP14-C8 and SP12-E10 gave EC_50_ values of 10.8 ± 1.4 nm and 1.75 ± 0.36 μm, respectively.

Single-cycle kinetic SPR analysis on a Biacore T200 of SP12-E10, SP14-C8, and SP17-F7 immobilized on a protein A surface using recombinant human extracellular domain of EpCAM as analyte gave affinities of 5.92, 0.76, and 58.9 nm, respectively (Table 1). The association rate constant (*K_a_*) of SP14-C8 (1.28 × 10^5^ 1/Ms) was significantly faster than for the other two antibodies, whereas the dissociation rate constants (*K_d_*) between all three antibodies varied by less than 2-fold.

**Table 1:** Summary of the antibody affinities measured by SPR and EC_50_ determination NA, not available.

We repeated the single-cycle kinetic SPR analysis by immobilization of the soluble EpCAM extracellular domain via its C-terminal His_6_ tag with the SP12-E10, SP14-C8, and SP17-F7 mAbs acting as the analytes (Fig. S7). In this case we only observed 17–18% of the expected activity for SP14-C8, only 4% of the expected activity for SP12-E10, and no binding activity observed for SP17-F7. Possibly indicating that only a fraction of the EpCAM extracellular domain or antibody was active on the anti-His tag capture surface, or it binds through a different stoichiometry, or this may be due to a preferred orientation of the EpCAM extracellular domain presented on the capture surface. However, affinities for both SP14-C8 and SP12-E10 could be determined, providing values of 0.26 and 380 nm, respectively. The large difference between the two SPR experiments for SP12-E10 appeared to be partly caused by a large shift in the observed *K_a_* from 9.5 × 10^3^ 1/Ms to 9.5 × 10^2^ 1/Ms.

Therefore, using our mammalian display technique, we have isolated an anti-EpCAM specific antibody with an apparent subnanomolar affinity. The observed difference between the measured EC_50_ and SPR values may be due to the diverse conditions of the two experiments, such as the high concentration of the purified soluble domain of EpCAM used in SPR experiment inducing an additional avidity effect. This contrasts with the lower recombinant levels of cell surface expression on the CHO cells used in the EC_50_ assay.

### Generation of CAR-T constructs with EpCAM scFv and CAR-T cell assays

Given the great interest in therapeutic immunomodulation and targeting of tumor cells with autologous lymphocytes genetically manipulated to express an scFv-based chimeric antigen T cell receptor (21), CAR-T cells were tested with our panel of three EpCAM binders. Two EpCAM-positive cell lines, MCF-7 (a breast cancer cell line) and the CHO–EpCAM cell line used for antibody selections, were used as targets and both Jurkat cells and CD3+ T cells derived from human peripheral blood mononuclear cells (PBMCs) were used as effector cells. CAR-T cells were generated by lentiviral transduction of either Jurkat cells or CD3+ T cells derived from human PBMC. Lentivirus was engineered to encode a second-generation CAR scaffold with anti-EpCAM scFv fused to a CD8-derived transmembrane region followed by a 4-1BB co-stimulatory domain and a CD3ζ chain.

**Figure 6:**
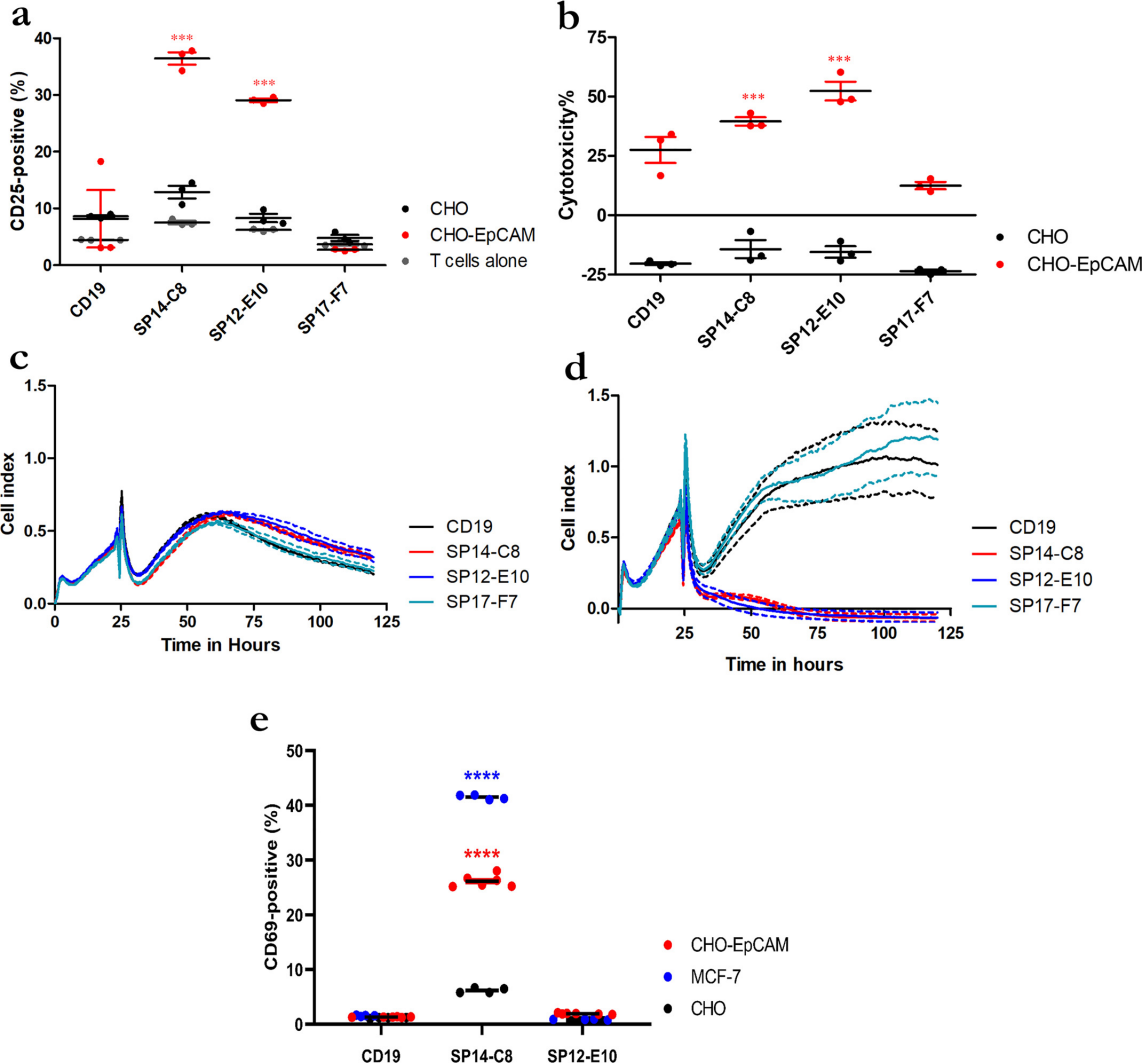
**Assessment of antigen specificity of CAR-T cells generated with the three selected antibodies.***a*, induction of activation marker CD25 on PBMC-derived CAR-T cells in co-culture with CHO–EpCAM or CHO-X cells (co-cultured at a ratio of 5:1) as measured by flow cytometry after 48 h of culture. *b*, cytotoxicity of PBMC-derived CAR-T cells when co-cultured with CHO-X or CHO–EpCAM as assessed by release of LDH after 48 h of culture. *c* and *d*, xCELLigence analysis of CHO-X (*c*) or CHO–EpCAM (*d*) cells co-cultured with PBMC-derived CAR-T cells (at a ratio of 1:5) monitored over 120 h. The data were measured in three biological triplicates where the mean is represented in *solid line* with the standard error shown by the *dotted line* of the same color. Statistical analysis was performed by two-way analysis of variance with Bonferroni post hoc tests and significance were assessed *versus* T cell cultured alone or CHO cells. ***, *P* < 0.001. *e*, assessment of Jurkat CAR-T cell activation by flow cytometry analysis of induction of activation marker CD69 after 4 h of co-culture with CHO–EpCAM, MCF7, or CHO-X cells (at a ratio of 1:1). The graph shows representative data from three independent experiments and shows the means and S.E. Statistical analysis was performed by two-way analysis of variance using Tukey's multiple comparison test for CHO–EpCAM or MCF7 cells compared with CHO-X cells. ****, *P* < 0.0001.

Transduced CD3+ T cells derived from human PBMC were expanded for 10 days before testing. Control CAR-T cells with the same architecture and an scFv recognizing CD19 were also generated. The antigen specificity of the CAR-T cells was tested in a co-culture with CHO-X and CHO–EpCAM cell lines. EpCAM–CAR-T cells displayed a significant increase in the expression of activation marker CD25 (36.5 and 29% by SP14-C8 and SP12-E10, respectively; Fig. 6*a*) and elicited cell cytotoxicity (40 and 52% by SP14-C8 and SP12-E10, respectively; Fig. 6*b*) only upon co-culture with target CHO–EpCAM cells and not with parental CHO-X cells. The control CD19–CAR-T cells did not display activation because CD25 expression was similar to the background level observed with T cells alone. An increase in the cytotoxicity against CHO–EpCAM cells was, however, observed (27%; Fig. 6*b*), suggesting a degree of cross-reactivity. We further explored the CAR-T cell–induced target cell killing in co-cultures of CHO-X and CHO–EpCAM cells measured in real-time by cell index, a unitless measure of cell viability. In the presence of control CD19–CAR-T cells, CHO-X and CHO–EpCAM cells persisted for 120 h (Fig. 6*c*), whereas with EpCAM–CAR-T cells (SP14-C8 and SP12-E10), complete lysis of CHO–EpCAM cells was observed within 30 h (Fig. 6*d*). No cell cytotoxicity was observed either with EpCAM–CAR-T cells (SP14-C8 and SP12-E10) in parental CHO-X cells or with CD19–CAR-T cells with either cell line.

Finally, the anti-EpCAM scFv CAR constructs were tested against MCF7 and CHO–EpCAM cells using lentivirus-transduced Jurkat cells as effectors. CAR-transduced Jurkat cells were generated with the same SP14-C8, SP12-E10, and CD19 lentiviral constructs as above to assess activation upon co-culture with EpCAM-expressing target cells. CAR Jurkat cells were incubated with parental CHO-X, CHO–EpCAM, and MCF7 cells for 4 h before assessing Jurkat cell activation via CD69 expression. Only the SP14-C8 CAR construct induced a significant level of CD69 in the presence of both CHO–EpCAM and MCF7 target cells compared with parental CHO-X cells (Fig. 6*e*). The lack of SP12-E10–mediated activation may be attributed to the temporal dynamics of CD69 expression, a transient early marker of activation, in addition to its observed lower affinity to EpCAM. These observations suggest that both SP14-C8 and SP12-E10 EpCAM CAR-T cells are functionally active and exhibit antigen-specific activation and cytotoxicity and that SP14-C8 is more potent than SP12-E10, consistent with its higher affinity.

## Discussion

Our approach has successfully identified scFvs against EpCAM using a naïve library that mimics the natural naïve human repertoire. This was achieved completely in a mammalian CHO cell line with no requirement for performing initial panning steps in other systems. This has the potential to be exploited as a novel approach to identifying new therapeutic antibodies against other therapeutic membrane proteins in their native environment *in vitro* and in a mammalian cell system routinely used for antibody expression and manufacture, thus ensuring the compatibility of the discovered antibodies with these systems.

The use of lentiviruses enabled transduction of large numbers of mammalian cells, and their preference for regions of open chromatin (22) was expected to ensure that the majority of the library was sufficiently expressed for detection of self-labeling. The dynamics of the transduction (*i.e.* the number of cells transduced by none, one, or more particles) were expected to follow a Poisson distribution and using a MOI of 1.0 theoretically resulted in 37% of cells with no events, 37% cells with one integration event, 18% cells with two integration events, and 8% cells with more than two integration events. In the current selection campaign described in this work, we did not isolate any cells with more than one integration event; however, in other campaigns (data not shown), we have isolated positive binders from cells with two integration events where both scFvs were expressed. This required resolution of the correct binder, which did not overly add to the complexity of the process, although resolving a situation with more than two integration events would be more challenging; because the frequency of more than two integration events is relatively low, we do not expect this to be problematic for selection campaigns. Furthermore, we are working to fine-tune the MOI to define the condition that provides the best compromise between the number of multiple integration events, the number of cells with no integration event and the total number of cells that can be successfully handled for the first selection step. In this work, the initial selection step was done with 4 × 10^9^ cells, and we believe that this can be routinely increased to at least 10^10^ with the implementation of further automation.

The major challenges of this approach lie in the potential for cross-labeling (where antibody secreted by one cell binds to the surface of another cell) and off-target labeling (where scFv specific for CHO cell surface proteins self-label or cross-label). We do not believe that cross-labeling is significantly problematic during early selections because the numbers of antigen-positive cells in the initial population of cells is extremely low, but the significance of the problem increases as the proportion of antigen-positive cells increases during selection. Therefore, we implemented two strategies to mitigate against these issues: the use of decoy cells expressing antigen only to reduce cross-labeling, combined with FRET as a method for reducing off-target labeling. Of the 93 clones selected after a second round of FRET-based cell sorting, 66 clones secreted an scFv that appeared to bind to the CHO-X parental cell line, whereas 26 were found to give an increased self-labeling signal in the presence of cell surface EpCAM (Fig. S2). Only one of the clones secreted an scFv that did not bind either to CHO-X or CHO–EpCAM cells, which suggested that cross-labeling had been effectively suppressed. In contrast, the FRET method to reduce the selection of off-target binders appears to have been only moderately successful, and there is still room for improvement for the avoidance of selection of cells secreting an off-target binder. Potentially, improving the intensity of the FRET signal and using a more stringent FACS-gating strategy will reduce the number of off-target binders. Nevertheless, the current rate of selection of off-target labeled cells is manageable. The FRET approach described herein also employed the use of a second antibody that was already known to bind the target; this may not be possible in instances in which the target is particularly challenging. However, the genetic addition of an established epitope tag has been successfully used as an alternative strategy.

One further issue we encountered was the selection of three antibodies that appeared to bind strongly to CHO–EpCAM cells with no signal against CHO-X cells (Fig. S3); one antibody in particular was heavily selected for. When these antibodies were recloned as scFv, they bound weakly to CHO–EpCAM cells but did not bind when expressed as whole IgG. The reasons for this are currently unclear.

Although the final number of initial antigen-positive cells selected was low (26), this was primarily caused by low efficiency outgrowth of single-cell clones following FACS, which was as low as 20% (data not shown). Improvements in cell outgrowth and increasing the numbers of cells sorted will increase the chances of selecting true antigen-positive cells and increase the diversity of sequences selected for. Further improvements in library design and creation are also required to increase the probability of selecting diverse sequences; the current library had a maximum theoretical diversity of ∼8.0 × 10^8^, whereas the number of independent clones was calculated to be ∼6 × 10^8^. As mentioned above, improvements in cell handling should allow us to transduce ∼10^10^ cells with Poisson statistics dictating that ∼5.5 × 10^9^ cells will contain one or two integration events; thus, interrogation of libraries of at least 10^9^ is feasible. Although this is not as large as some phage display libraries that have been created (23, 24, 25)^,^ our goal has been to create libraries that contain a high proportion of functional sequences that are designed to avoid sequence motifs that may be problematic during manufacturing. Despite these limitations, we were able to isolate a specific EpCAM binder with subnanomolar affinity.

We believe that this system has a particular utility for identifying scFv binders against membrane proteins for CAR-T cell applications. To this end, we reformatted the top three binders as second-generation CARs, which were transduced into both CD3+ T cells isolated from human PBMC and Jurkat cells. We were able to demonstrate that the two higher-affinity scFv CAR constructs (SP14-C8 and SP12-E10) were capable of triggering antigen-specific activation of the primary T cells and directing potent cytotoxicity against CHO–EpCAM cells. SP14-C8 CAR-transduced Jurkat cells were also potently activated in the presence of an antigen-positive MCF-7 breast cancer cell line and CHO–EpCAM cells, although the SP12-E10 CAR was not active in this assay, possibly due to the activation kinetics of the cells, the constraints imposed by a CAR-T configuration compared with that of a soluble scFv, or the lower affinity of this antibody.

In conclusion, we believe that the novel display method reported here is the first platform enabling the discovery of target specific scFvs from an untrained library in a fully mammalian cell system. This platform offers the opportunity to identify novel antibodies against membrane proteins in a physiological setting, with desirable properties for use either in a CAR-T cell setting or as a whole mAb therapy.

## Materials and methods

### Construct design

A self-inactivating pSF lentivector was modified to accommodate subcloning of PciI/XbaI EpCAM DNA (accession no. BC014785.1) fragments. The pSF lentivector contains a cytomegalovirus promoter with TetO sites that drives the expression of the transgene as well as a TetR gene via an spleen focus forming virus promoter downstream of the transgene, allowing control of the target protein by doxycycline. A puromycin resistance gene was incorporated following the TetR gene and separated by an encephalomyocarditis virus (EMCV) IRES sequence to enable antibiotic selection following transduction. Finally, the woodchuck hepatitis virus post-transcriptional regulatory element was incorporated downstream of the puromycin resistance gene.

For the scFv library construction, a self-inactivating pSF lentivector was modified to accommodate subcloning of KpnI/XhoI scFv DNA fragments. The pSF lentivector has a spleen focus forming virus promoter that drives the expression of the transgene followed by the woodchuck hepatitis virus post-transcriptional regulatory element downstream. The EpCAM-binding scFv-positive control was derived from sequence 75 from U.S. patent 7435549. The CHESS scFv library was constructed by randomly combining all known functional human germline V, D, and J segments that had been synthesized *de novo* (TWIST Bioscience, San Francisco, CA. USA). Human germline V_κ_ and V_λ_ elements flanked by SpeI/SapI restriction sites were ligated to human germline J_κ_ and J_λ_ elements flanked by SapI/PciI restriction sites. A signal sequence was incorporated upstream of the light chain segment and a 15-amino acid GS-linker downstream. The heavy chains were constructed from all the known human germline V_H_ sequences flanked by XbaI and SapI restriction sites, which were ligated to the D and J_H_ segments that had been constructed from an oligonucleotide pool annealed and PCR-amplified with adapter sequences flanked by SapI/XhoI restriction sites. Nucleotides were added to the 5′ ends of the D segments to ensure representation of all three reading frames. All assembled segments were transformed via electroporation into competent *Escherichia coli* (MC1061 F− electrocompetent cells; Cambridge Bioscience, catalog no. 60514-2) and titred to determine the number of individual transformants. We are willing to supply this CHESS scFv library described to anyone who requests it for a stated purpose, subject to a material transfer agreement.

### Next-generation sequence analysis of scFv library

Library amplicons were sequenced by GENEWIZ using paired-end reads on an Illumina based platform. Next-generation sequencing (NGS) reads were processed using the Immcantation pipeline (26) alongside custom R packages. IgBLAST (27) was used to perform antibody-based annotation on NGS reads. The Immcantation R package, Alakazam (28), was used to load tabulated NGS data into R, and the composition of the CDR regions within the scFv library population was determined. Sequence logos and histograms were produced using ggseqlogo and ggplot2, respectively (29, 30).

### Cell lines

OXGENE in-house CHO cells (CHO-X) were maintained and expanded for experiments in complete growth medium containing ProCHO 5 (Lonza, catalog no. BE12-766Q) supplemented with 4 mm UltraGlutamine (Lonza, catalog no. BE17-605E/U1) in shaking flasks (Corning) at 37 °C under 5% CO_2_ (125 rpm in a Climo-Shaker ISF1-XC incubator, Kuhner AG).

A CHO-X cell pool stably expressing full-length EpCAM was initially generated by transduction of CHO-X at a density of 0.3 × 10^6^ cells/ml with a lentivirus packaging the EpCAM gene and a puromycin-resistant marker at MOI of 2.5. 72 h after transduction, the medium was replaced, and the cells were further cultured in medium containing puromycin (3 µg/ml) for 14 days. After recovery and expansion of the pool, clonal cell lines were generated by cell surface staining with an EpCAM FITC antibody (BD Biosciences, catalog no. 347197) and single-cell sorting on a SH800 Cell Sorter (Sony). The 64% highest-expressing cells were single cell sorted into 96-well plates. The clones were manually picked on day 18 and expanded. CHO clones were screened for EpCAM expression by flow cytometry 35 days after sorting, and clone SP1D12 was chosen for further work based on EpCAM expression levels.

Adherent HEK-293T cells European Collection of Authenticated Cell Cultures (ECACC) were grown in Dulbecco's modified Eagle's medium (Sigma–Aldrich, catalog no. D5796) supplemented with 10% FBS (Thermo Fisher Scientific, catalog no. 10500-064) in a static incubator (CB 160, Binder) at 37 °C under 5% CO_2_. In-house generated HEK-293 EBNA suspension adapted cells (293OX-EBNA) were grown in BalanCD HEK293 (Irvine Scientific, catalog no. 91165) supplemented with 4 mm UltraGlutamine at 37 °C under 8% CO_2_ (125 rpm in a Climo-Shaker ISF1-XC incubator).

Jurkat cells (CLS) were maintained in RPMI 1640 (Sigma, catalog no. R8758) supplemented with 10% FBS in a static incubator (CB 160, Binder) at 37 °C under 5% CO_2_. MCF-7 cells were maintained in minimal essential medium (Sigma–Aldrich, catalog no. M4655), 2 mm GlutaMAX (Thermo Fisher Scientific, catalog no. 35050061), sodium pyruvate (Thermo Fisher Scientific, catalog no. 11360-070), nonessential amino acids (Thermo Fisher Scientific, catalog no. 11140035), and 10% FBS (Thermo Fisher Scientific, catalog no. 10500-064) in a static incubator (CB 160, Binder) at 37 °C under 5% CO_2_.

### Lentiviral preparation

The CHESS scFv synthetic library was packaged into replication-incompetent lentiviral particles in HEK-293T cells. 2 × 10^7^ cells were seeded 3 days before transfection in hyperflasks (Corning, catalog no. 10020). The cells were transfected with 760 µg of total DNA (CHESS library and lentiviral packaging vectors) and 1900 µg of branched polyethyleneimine transfection reagent (Sigma–Aldrich, catalog no. 408727). The resulting lentiviral supernatants were harvested 3 days later, clarified by centrifugation at 800 × *g* for 10 min, and stored frozen at −80 °C. The physical titer of the lentiviral preparations was determined by quantitative PCR, and the infectious titer on CHO–EpCAM cells of lentiviruses encoding GFP was determined by flow cytometry. The infectious titer of the library lentiviral preparation was calculated by using the physical/infectious titer ratio determined for the GFP lentiviruses.

### Library transduction

1.35 × 10^9^ CHO–EpCAM cells adjusted to a cell density of 0.9 × 10^6^ cells/ml were transduced with lentiviral particles packaging the scFv library at MOI = 1. Cell culture medium was then supplemented with 1 µg/ml doxycycline (Sigma–Aldrich, catalog no. D3072) to induce EpCAM expression. After 3 days the medium was changed, and the cells were incubated overnight at 25 °C in culture medium with 5 µg/ml doxycycline before magnetic selection (MACS).

### Magnetic activated cell sorting

MACS was done using an autoMACS Pro Separator (Miltenyi Biotec) according to the manufacturer's instructions. MACS buffer consisting of PBS, pH 7.4 (Thermo Fisher Scientific, catalog no. 14190-094), with 10% FBS and 2 mm EDTA (Sigma–Aldrich, catalog no. 03690) was used throughout the process. For the first round of selection, 4 × 10^9^ CHO–EpCAM cells transduced with the lentiviral library were labeled with 400 μl of PE-conjugated anti-HA antibody (1 μl/10 × 10^6^ cells, Miltenyi Biotec, catalog no. 130-120-717) for 30 min at 4 °C followed by labeling with 4 ml of anti-PE microbeads (10 μl/10 × 10^6^ cells, Miltenyi Biotec, catalog no. 130-048-801) for 25 min at 4 °C and subsequent positive cell selection using the AutoMACS instrument (program Depl025). In subsequent rounds, the process was repeated with quantities of detection reagents adjusted for the input quantity of cells.

Between rounds, the enriched fraction of cells was allowed to recover for 3 days before reinduction with 1 µg/ml doxycycline for a further 3 days. The culture medium containing doxycycline was then replaced, and cells were incubated overnight at 25 °C prior to the subsequent sort.

### FACS selection of FRET-positive cells

CHO–EpCAM cells were labeled with CellTracker^TM^ Blue CMAC dye (Thermo Fisher Scientific, catalog no. C2110) and added in excess (9:1) to either the output from the third round of MACS selection or the output from the first FACS pool sort. Briefly, 5 × 10^7^ cells were centrifuged (300 × *g* for 5 min) and resuspended in PBS, pH 7.4, containing 1% BSA and 150 μm CMAC. The cell suspension was incubated for 40 min at 37 °C in a shaking incubator for intracellular labeling. CMAC-labeled cells were washed once with PBS, pH 7.4, 1% BSA to remove excess dye. Cell suspensions of capturing decoy cells, as well as the cell sample to sort were prepared at 0.4 × 10^6^ cells/ml in culture medium containing doxycycline.

FACS on these induced cells was performed using an SH800 cell sorter (Sony) with Cell Sorter software (Sony). In the first round of FACS, 5 × 10^7^ cells were washed once with culture medium containing 2 mm EDTA and stained in the same medium with 5 nm anti-HA-PE antibody (Miltenyi Biotec) and 30 nm anti-EpCAM–Alexa Fluor 647 antibody (Biolegend, catalog no. 324212) for 30 min at 4 °C. Control cells were single stained with each antibody separately under the same conditions. The cells were washed with culture medium containing EDTA and finally resuspended in the same buffer for analysis and sorting. Live cells were gated based on scatter properties; for simplicity a cell viability dye was not used. The sort gate was drawn on a bidimensional dot plot showing the emission signal of Alexa Fluor 647 *versus* PE.

For the first sort, 36,000 cells were sorted into 50% conditioned growth medium, 2.5% ClonaCell^TM^–CHO ACF supplement (STEMCELL Technologies, catalog no. 03820), 40% fresh growth medium and allowed to recover and expand. A second round of FRET selection was performed following the same protocol except that MACS buffer was used throughout the process, and the cell suspension was sorted into single cells in 75% conditioned growth medium, 2.5% ClonaCell^TM^, 15% fresh growth medium. The cells were expanded and supplemented with 50% fresh medium after 2 weeks, followed by transfer to fresh growth medium after about 3 weeks.

### Flow cytometry analysis

Flow cytometry immunostaining experiments were performed using an Attune^TM^ NxT flow cytometer (Thermo Fisher Scientific) equipped with two lasers of 488 and 561 nm, connected to an Attune^TM^ NxT acoustic focusing cytometer autosampler (Thermo Fisher Scientific). The samples were stained in and sampled from U-bottom 96-well plates (Corning). The cell samples were harvested and washed once with MACS buffer before incubation for 30 min at 4 °C with the corresponding antibody diluted in MACS buffer. The cells were washed twice with the same buffer before final resuspension. The cells were gated based on scatter properties, and doublets were excluded by plotting FSC-H *versus* FSC-A. At least 10,000 events on the single cells gate were acquired. Flow cytometry data were analyzed using Attune^TM^ NxT software (Thermo Fisher Scientific).

### Expression of recovered scFv and whole IgG

ScFvs isolated from clonal CHO–EpCAM cells were recovered by PCR and recloned either as scFv into the lentiviral expression plasmid as described above or as whole IgG1 into a dual expression vector, where the heavy and light chains were expressed from separate gene cassettes on the same plasmid, which also contained an EBV OriP element. Plasmid DNA was prepared and used to transfect suspension 293OX-EBNA cells using FectoPro (Polyplus, catalog no. 116-001) according to the manufacturer's instructions in a volume of either 4 ml for supernatant analysis or 200 ml for purification of whole IgG. Protein A purification was done using 1 ml of HiTrap MabSelect SuRe columns (GE Healthcare, catalog no. 11003493) according to the manufacturer's instructions. Purified antibodies were desalted using Zeba Spin columns (Thermo Fisher Scientific, catalog no. 89891) and quantified by absorbance at 280 nm.

### CHO–EpCAM cell binding of scFv and whole IgG1 antibodies derived from anti-EpCAM hits

The supernatants from either scFv or whole IgG transfections were incubated with either control CHO-X cells or CHO–EpCAM cells for 1 h at 37 °C in a shaking incubator and then were washed twice with MACS buffer before resuspension and incubation with either anti-HA–PE or anti-Fc–PE at 4 °C for 30 min. Finally, the cells were washed a further two times before analysis on an Attune NxT flow cytometer.

For the EC_50_ determination, titrations (0.1 nm to 10 μm in doubling dilutions) of purified whole IgG1 antibodies derived from the clones SP14-C8, SP17-F7, and SP12-E10 were applied to either CHO-X control cells or CHO–EpCAM cells and incubated for 1 h at 37 °C in a shaking incubator and then washed twice with MACS buffer before resuspension and incubation with anti-Fc–PE (Biolegend, catalog no. 409304) at 4 °C for 30 min. Finally, the cells were washed a further two times before analysis on an Attune NxT flow cytometer. The cells were gated based on scatter properties, and doublets were excluded by plotting FSC-H *versus* FSC-A. At least 10,000 events on the single cells gate were acquired. Flow cytometry data were analyzed using Attune^TM^ NxT software.

### Affinity measurements using surface plasmon resonance

The affinity of purified whole IgG EpCAM-positive antibody variants for the EpCAM ECD was determined by surface plasmon resonance (SPR) measurements on a Biacore T200 instrument (GE Healthcare). Each of the anti-EPCAM antibody variants was immobilized onto the surface of a protein A sensor chip. The assays were run in PBST buffer (0.05% Tween 20, PBS, pH 7.4), and recombinant EpCAM-ECD (AcroBiosystems, catalog no. EPM-H5223) was assayed for binding at concentrations between 0.3 nm and 10 μm, followed by a single dissociation step (binding parameters were determined with the Langmuir single binding site model using Biacore T200 evaluation software 2.0.3 (GE Healthcare)).

The second SPR experiment was performed by EpCAM-ECD (AcroBiosystems, catalog no. EPM-H5223) immobilized onto the surface of a sensor chip by an anti–His tag capture antibody. The assays were run in PBST buffer (0.05% Tween 20, PBS, pH 7.4) and recombinant anti-EpCAM antibodies were assayed for binding at concentrations between 1 nm and 3.33 μm, followed by a single dissociation step (binding parameters were determined with the Langmuir single binding site model using Biacore T200 evaluation software 2.0.3 (GE Healthcare)).

### Processing of peripheral blood mononuclear cells and T-cell isolation

Human peripheral blood from anonymized healthy donors was obtained from the National Health Service Blood and Transfusion Service. PBMCs were purified by Ficoll (GE Healthcare, catalog no. 17144002) density gradient centrifugation as recommended by the manufacturer. For extraction of CD3-positive T cells from PBMC, non-CD3 cells were depleted using a Pan T cell isolation kit (Miltenyi Biotec, catalog no. 130-096-535). Isolated T cells were resuspended in RPMI 1640 (Gibco, catalog no. R8758) supplemented with 10% heat-inactivated fetal bovine serum, 2 mm l-glutamine (Gibco, catalog no. 25030-081), 10 mm HEPES (VWR Life Science, catalog no. J848), 0.5 mm sodium pyruvate (Sigma, catalog no. S8636), 1× nonessential amino acids (Gibco, catalog no. 11140-035), 100 units/ml penicillin, and 100 μg/ml streptomycin (Gibco, catalog no. 15140-122) (T-cell medium).

### CAR transduction and expansion of CD3 T cells

On day 0, T cells were resuspended in T-cell medium at 10^6^ cells/ml and dispensed at 100 μl/well into a 96-well plate. 2 μl/well CD3/CD28 Dynabeads (Thermo Fisher Scientific, catalog no. 111.61D) were washed following the manufacturer guidelines, resuspended in 50× volume T-cell medium containing 200 units/ml IL-2 (Miltenyi Biotec, catalog no. 130-097-746) and 20 ng/ml IL-7 (Miltenyi Biotec, catalog no. 130-095-362) and added to the 96-well plate at 100 μl/well. On day 1, 120 μl of medium was removed and replaced with lentiviruses encoding candidate CARs (MOI = 10). On day 5, the cells were transferred to a 24-well plate and cultured in cytokine supplemented T-cell medium until the cells were ∼90–95% confluent; thereafter cultures were scaled up to a T25 flask.

### Assessment of CAR-T cell activation and cytotoxicity during culture with target cells

Expanded CAR-T cells derived from two healthy donors were independently harvested, counted, and incubated with either CHO-X or CHO–EpCAM cells (target/effector ratio, 1:5). After 48 h, the cell samples were harvested and analyzed by flow cytometry to measure T-cell activation using an anti-CD25 antibody (Clone BC96, Biolegend, catalog no. 302606). Supernatants were also harvested, and total cytotoxicity was assessed by measuring release of lactate dehydrogenase using a CytoTox 96 nonradioactive cytotoxicity assay (Promega, catalog no. G1780)

### Analysis of time-dependent cytotoxicity using xCELLigence

Viability of CHO-X and CHO–EpCAM cells incubated with transduced CAR-T cells (ratio of 1:5) was monitored in real time using xCELLigence RTCA DP technology (Acea Biosciences). The cells were incubated for 24 h (37 °C, 5% CO_2_) before CAR-T cells were added, and cell impedance was measured every 15 min for a total of 120 h.

### CAR transduction and expansion of Jurkat cells

Jurkat cells were incubated in culture medium (RPMI 1640, 10% FBS) at 2 × 10^5^ cells/well with lentiviruses encoding candidate CARs at MOI = 20 (120 μl/well) in a round-bottomed 96-well plate (Corning). On the following day, 100 μl of culture medium was added to each well, and on day 3 the cells were transferred to a 48-well plate (final volume, 300 μl/well). On day 4, the cells were expanded by seeding at a density of 1 × 10^5^ cells/ml and dispensed into multiple wells of a 48-well plate. The cells were subsequently expanded in culture medium.

### Assessment of CAR Jurkat cell activation via CD69 expression

CAR Jurkat cell lines, CHO-X, CHO–EpCAM and MCF-7 cells were washed once in culture medium and adjusted to a density of 1 × 10^6^ cells/ml. CAR Jurkat cells were co-incubated with each target cell line at a ratio of 1:1 (200 μl/well, 96 round-bottomed plate) for 4 h at 37 °C. The cells were washed twice with MACS buffer and stained with anti-human CD69 FITC (BioLegend, catalog no. 310904) for 25 min at 4 °C. The cells were then washed three times with MACS buffer, followed by analysis of CD69 expression on an Attune^TM^ NxT flow cytometer (ThermoFisher Scientific) with accompanying software.

## Data availability

All data are contained within the article.
