# Supplementary material for: Development of a novel mammalian display system for selection of antibodies against membrane proteins
Source: J Biol Chem. 2021 Jan 13;295(52):18436–48. doi: 10.1074/jbc.RA120.015053 (PMC7939478; doi:10.1074/jbc.RA120.015053)
Supplement: Supplementary file 1 [file mmc1.pdf]

Development of a novel mammalian display system for selection of antibodies against membrane proteins

Nathan Robertson*[1], Nancy Lopez-Anton*[1], Shalom A. Gurjar*[1], Hena Khalique[2], Zainab Khalaf[1], Siobhan Clerkin[1], Vaughan R, Leydon[1], Richard Parker-Manuel[1], Alexander Raeside[1], Tom Payne[1], Tim D, Jones[1], Len Seymour[2] and Ryan Cawood†[1]

[1]OXGENE, Medawar Centre, Robert Robinson Avenue, Oxford, OX4 4HG, United Kingdom

[2]Anticancer viruses and cancer vaccines group, Department of Oncology, University of Oxford

*contributed equally to the work

†corresponding. rcawood@oxgene.com

Keywords: Mammalian display, Epithelial cell adhesion molecule (EpCAM), Chimeric antigen receptor T cells (CAR-T), therapeutic antibody discovery.

Supplementary information

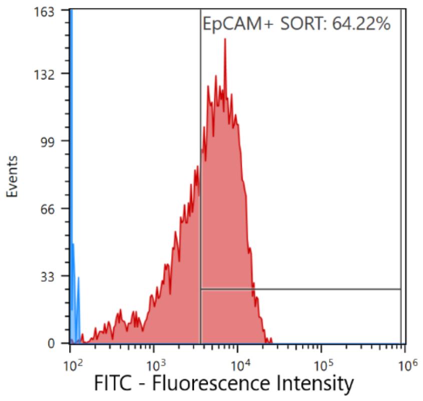

Supplementary Figure 1. Fluorescence activated cell sorting of the CHO-EpCAM pool. CHO cells transduced with a lentivirus encoding full-length EpCAM were selected with puromycin and induced with doxycycline to trigger target expression. Cells were either stained with an EpCAM-FITC antibody (red) or unstained (blue) and the 65% highest expressing cell population was sorted into single cells.

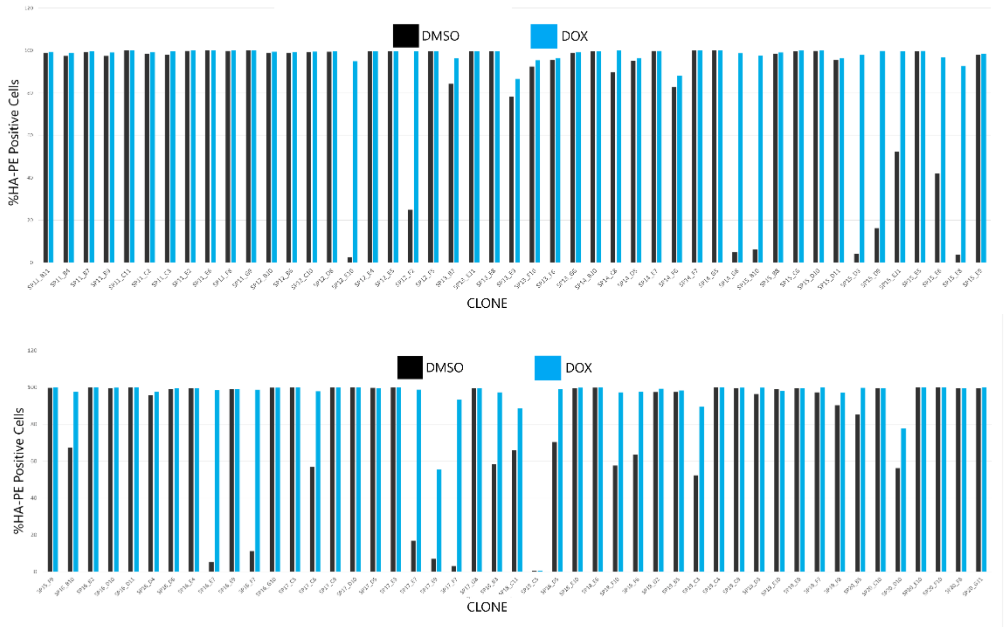

Supplementary Figure 2. Clonal expansions of single cells sorted by FACS based on the FRET signal were incubated either in the presence or absence of doxycycline and then stained with an anti-HA tag PE antibody, followed by flow cytometric analysis.

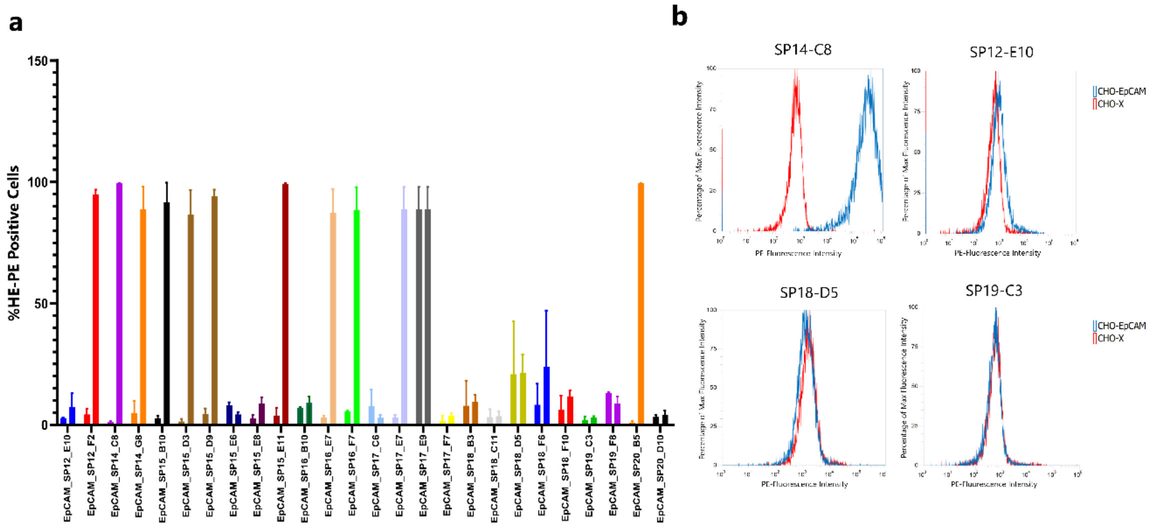

Supplementary Figure 3. Screening of supernatants from selected clones that showed differential self-labelling by flow cytometry. a) Supernatants were incubated with CHO-EpCAM cells or CHO-X control cells and stained with anti-HA PE (left bar CHO-X, right bar CHO-EpCAM). b) Examples of flow cytometry histograms of four clones exhibiting strong differential (SP14-C8), or weak differential (SP12-E10) signals, false binding (SP18-D5) or non-binding (SP19-C3).

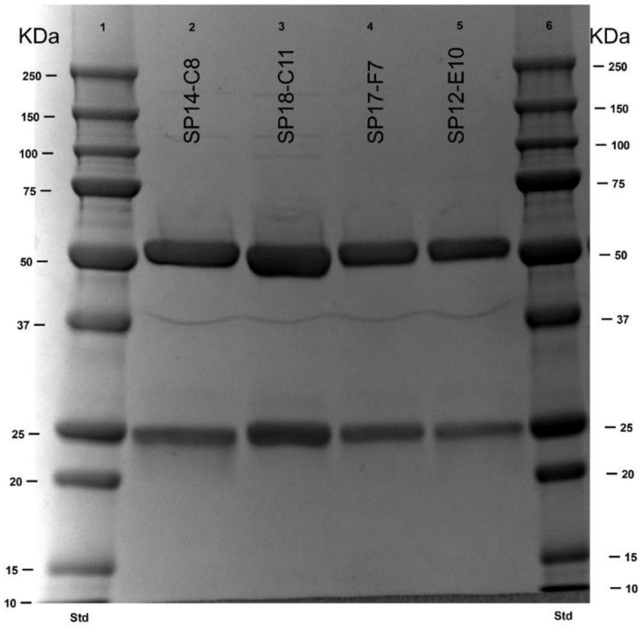

Supplementary Figure 4. Reducing SDS-PAGE gel (4-12% Tris-Glycine WedgeWell™, ThermoFisher Scientific, UK, Cat. No. 15446814) image for the purified anti-EpCAM candidates reformatted as full human IgG1/κ antibodies. Protein standards are Precision Plus Protein™ All Protein Standards (BIO-RAD, USA, Cat. No. 1610373) with sizes as indicated. The gel was stained with InstantBlue™ Protein Stain (Expedeon, U.S.A. Cat. No. ISB1L).

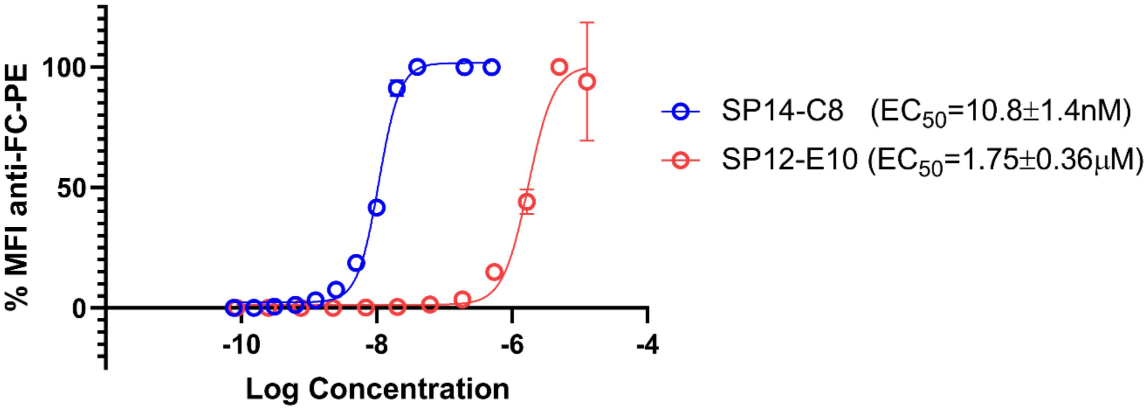

Supplementary Figure 5. Purified anti-EpCAM antibodies SP14-C8 and SP12-E10, re-formatted as whole IgG1, titrated against CHO-EpCAM and CHO-X control cells. Cells were stained with anti-Fc PE, analysed by flow cytometry and the MFI plotted against antibody concentration. The mean EC_{50} +/- standard error of mean in brackets are indicated. All data shown are representative of n=2 biologically independent experiments performed in duplicate.

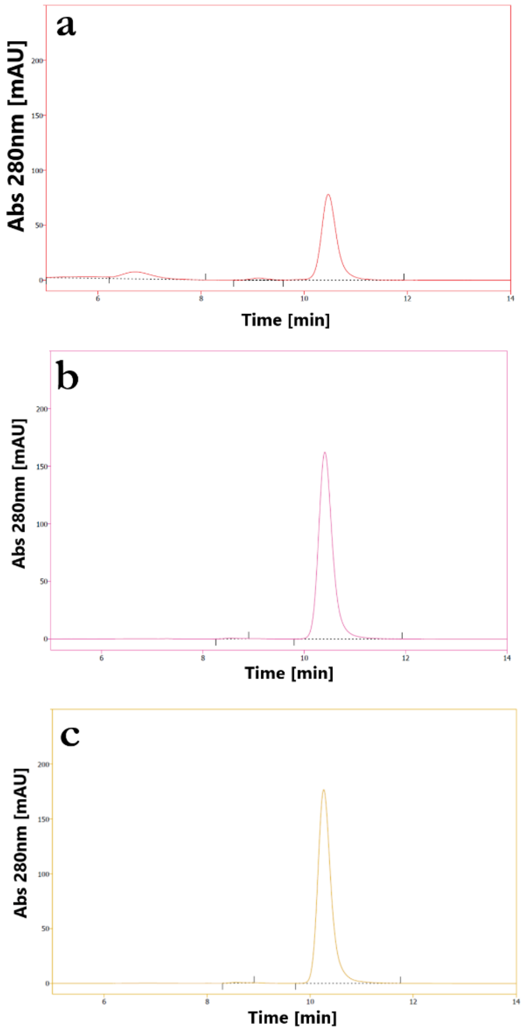

Supplementary Figure 6. Analytic HPLC-SEC of the three candidate anti-EpCAM antibodies reformatted as whole IgG1/κ and protein A purified. The antibodies were analysed using a TSKgel®SuperSW mAb HR (Tosoh, Japan, Cat. No. 0022854) gel filtration column on an Agilent 1200 LC system. a) SP14-C8 – The peak with the retention time of 10.467 min corresponds to monomeric mAb and represents 89.4% of the protein in the preparation. Three high-molecular weight species (HMWS1, HMWS2, and HMWS3), likely corresponding to aggregated protein, are seen as separate peaks and together make up the remaining protein in the preparation. b) SP17-F7 - The peak with the retention time of 10.407 min corresponds to monomeric mAb and represents 99.7% of the protein in the preparation. c) SP12-10 - The peak with the retention time of 10.26 min corresponds to monomeric mAb and represents 99.7% of the protein in the preparation.

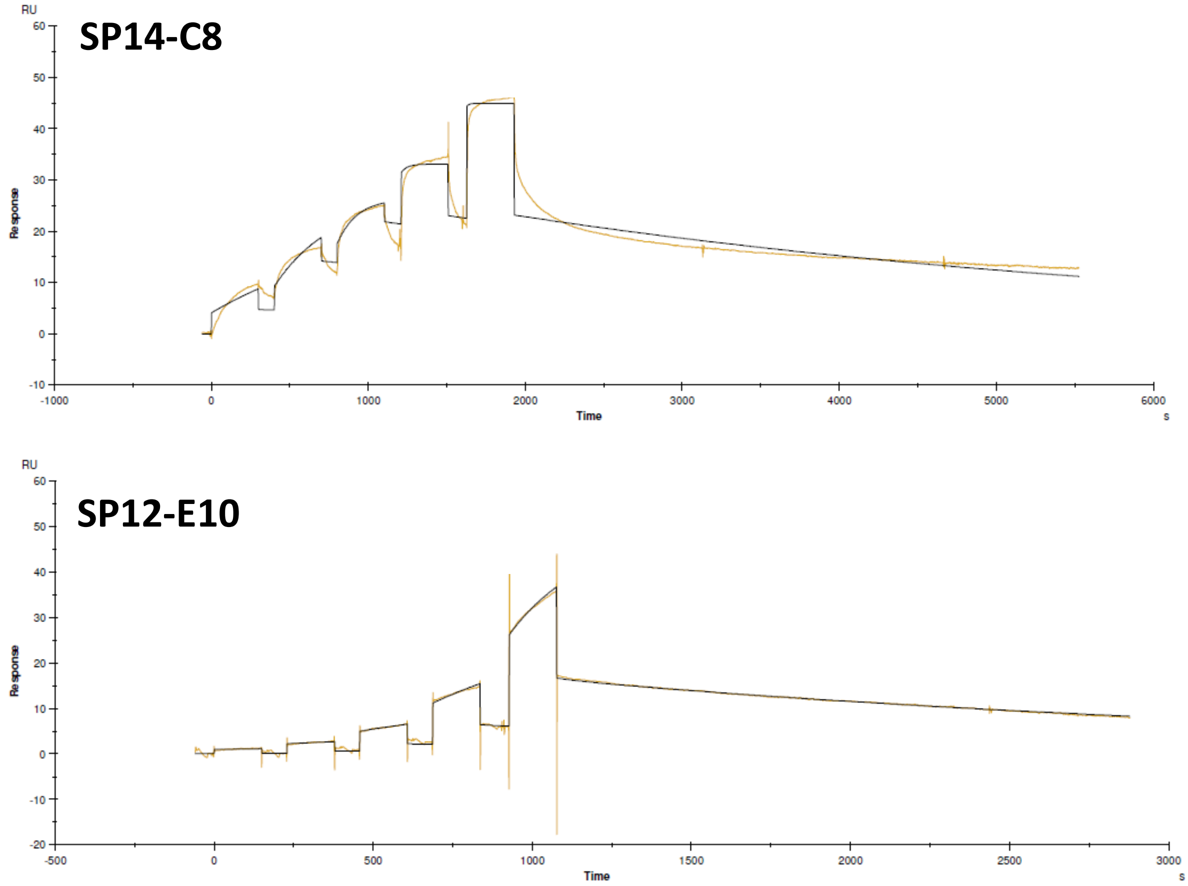

Variant	K_a (1/Ms)	K_d (1/s)	K_D (M)	Chi^2 (RU^2)
SP14-C8	7.87E+05	2.03E-04	2.59E-10	3.33
SP12-E10	1.02E+03	3.84E-04	3.78E-07	0.776

Supplementary Figure 7. Single-cycle kinetics SPR sensorgrams of EpCAM-ECD captured on anti-histag sensor chips binding to the mAbs SP12-E10 and SP14-C8. The data from the curve-fitting is shown in the table. SPR traces show representative data from six independent experiments.
